# Strengthening Health Systems in Humanitarian Settings: Multi-Stakeholder Insights on Contraception and Postabortion Care Programs in the Democratic Republic of Congo and Somalia

**DOI:** 10.3389/fgwh.2021.671058

**Published:** 2021-08-30

**Authors:** Nguyen Toan Tran, Janet Meyers, Bibiche Malilo, Julien Chabo, Jean-Baptiste Muselemu, Bienvenu Riziki, Patrick Libonga, Abdikani Shire, Hussein Had, Mohamed Ali, Mohamed Abdi Arab, Jama Mohamed Da'ar, Mohamed Hussein Kahow, Joseph Ege Adive, Binyam Gebru, Emily Monaghan, Catherine N. Morris, Meghan Gallagher, Virginie Jouanicot, Natacha Pougnier, Ribka Amsalu

**Affiliations:** ^1^Faculty of Health, Australian Centre for Public and Population Health Research, University of Technology Sydney, Sydney, NSW, Australia; ^2^Faculty of Medicine, University of Geneva, Geneva, Switzerland; ^3^Save the Children, Washington, DC, United States; ^4^Save the Children International Democratic Republic of Congo (DRC), Goma, Democratic Republic of Congo; ^5^Save the Children International Somalia, Gardo, Somalia; ^6^Save the Children UK, London, United Kingdom; ^7^Preterm Birth Initiative, University of California, San Francisco, San Francisco, CA, United States

**Keywords:** health system strengthening, family planning, contraception, postabortion care, ownership, accountability, Somalia, Democratic Republic Congo

## Abstract

**Background:** In humanitarian settings, strengthening health systems while responding to the health needs of crisis-affected populations is challenging and marked with evidence gaps. Drawing from a decade of family planning and postabortion care programming in humanitarian settings, this paper aims to identify strategic components that contribute to health system strengthening in such contexts.

**Materials and Methods:** A diverse range of key informants from North Kivu (Democratic Republic of Congo, DRC) and Puntland (Somalia), including female and male community members, adolescents and adults, healthcare providers, government and community leaders, participated in qualitative interviews, which applied the World Health Organization health system building blocks framework. Data were thematically analyzed according to this framework.

**Results:** Findings from the focus group discussions (11 in DRC, 7 in Somalia) and key informant interviews (seven in DRC, four in Somalia) involving in total 54 female and 72 male participants across both countries indicate that health programs in humanitarian settings, such as Save the Children's initiative on family planning and postabortion care, could contribute to strengthening health systems by positively influencing national policies and guidance, strengthening local coordination mechanisms, capacitating the healthcare workforce with competency-based training and supportive supervision (benefiting facilities supported by the project and beyond), developing the capacity of Ministry of Health staff in the effective management of the supply chain, actively and creatively mobilizing the community to raise awareness and create demand, and providing quality and affordable services. Financial sustainability is challenged by the chronically limited healthcare expenditure experienced in both humanitarian contexts.

**Conclusions:** In humanitarian settings, carefully designed healthcare interventions, such as those that address the family planning and postabortion care needs of crisis-affected populations, have the potential not only to increase access to essential services but also contribute to strengthening several components of the health system while increasing the government capacity, ownership, and accountability.

## Introduction

Sixty-one percent of maternal deaths worldwide occur in countries affected by fragility and crisis (1). In Somalia and the eastern region of the Democratic Republic of Congo (DRC), decades of unrest, fragility, and lack of financial investment have affected the health system with dire health consequences, including sexual, reproductive, maternal, newborn, child, and adolescent health (2, 3). Maternal mortality ratios in both countries remain among the highest in the world, although they have decreased over the past decade. In DRC, there were 473 maternal deaths per 100,000 live births in 2017 down from 542 in 2010 (1), and in Somalia, there were 692 maternal deaths per 100,000 live births in 2020 down from 985 in 2010 (4).

One of the leading causes of maternal mortality and morbidity is unsafe abortion, to which 4.7-13.2% of maternal deaths are attributed worldwide (5). South Asia and sub-Saharan Africa account for an overwhelming majority of these deaths (6). Additionally, women with an unmet need for modern contraception account for 84% of all unintended pregnancies in low-income countries (the highest proportion of women with an unmet need is in Sub-Saharan Africa at 21%) (7). Such a high level of unmet need contrasts with the established evidence that increased contraceptive use contributes to reduced maternal mortality (8).

In 2011, in partnership with the national Ministries of Health (MoH) and local health authorities, Save the Children started implementing family planning (FP) and postabortion care (PAC) services in diverse humanitarian settings to address the unmet need for contraceptives and treat the complications of miscarriage or induced abortion. PAC services included counseling, treatment of incomplete abortion, unsafe abortion and other complications, postabortion contraception to prevent unplanned pregnancies or practice birth spacing, linkage to reproductive and other health services, and community and service-provider partnerships (9). Access to contraceptive services is essential to prevent unsafe abortion in humanitarian settings, where damaged infrastructure, forced displacement, and security risks can further compromise access to quality PAC services (10). Therefore, FP was not only provided in the context of PAC but also as a standalone service to enable all women to prevent and space their pregnancies as they desired. Both FP and PAC are integral parts of the Minimum Initial Service Package (MISP) for sexual and reproductive health (SRH) in humanitarian settings (other MISP components comprise preventing sexual violence and responding to survivors' needs, preventing the transmission of HIV and other sexually-transmitted infections, preventing excess maternal and neonatal deaths and illnesses, ensuring the availability of safe abortion care to the full extent of the law, in addition to ensuring effective coordination and planning for comprehensive SRH services) (11).

The program strategy aimed to address the main barriers to FP and PAC found in each country. Barriers comprised healthcare providers' insufficient knowledge, limiting attitudes, and lack of skills based on the latest evidence, inadequate coverage of FP and PAC services, with PAC services mostly available in referral hospitals and relying on dilatation and curettage, low demand for FP and PAC services, unmet demand for FP and PAC services and restricted availability of supplies (12). In response to these barriers, Save the Children, in partnership with the respective Ministries of Health, developed a multi-pronged intervention model strategy to enhance access to and uptake of quality FP and PAC services. On the demand side, the model comprised community collaboration and mobilization to improve knowledge, change attitudes, generate demand, and ensure shared accountability. On the supply side, the main components to strengthen the quality of services were training and mentoring of service providers in addition to topping up low salaries; building the capacity of MoH staff; securing commodities, and supplies; renovations to create private and confidential spaces for services; and managing data for ongoing monitoring, evaluation, decision-making, and action in partnership with the MoH, providers, and community stakeholders.

The program underwent periodic monitoring and evaluation reviews, including client satisfaction surveys, register reviews, and scientific studies, which were presented at international venues, published in peer-reviewed journals, or both (12–17). Results from these studies showed the overall feasibility of FP and PAC programs in mostly protracted crisis humanitarian settings (with an acute phase due to the Ebola outbreak in the DRC), a high level of acceptability by clients above 90% (14), and the shift from the harmful practice of dilatation and curettage to manual vacuum aspiration or medication use for the provision of PAC (12). The increased uptake and 12-month continuation of modern contraceptives (pills, injectables, implants, and intrauterine devices) in standalone, postabortion (70% in DRC and 82% in Somalia), and postpartum services with greater use of long-acting methods (implants and intra-uterine devices) was a noticeable outcome (12, 13, 18).

How to respond to the health needs of populations affected by humanitarian crises with programs that can also contribute to long-term development epitomizes the tensions within the humanitarian-development nexus (19). Such pressures may stem from differences in organizational cultures, mandates, and principles between humanitarian and development (20). Expectations that strengthened health systems should be de facto sustainable may compound such tensions (21). Despite a wealth of programmatic experience from the field, a 2018 literature review on health system strengthening and coordination in countries under stress showed limited evidence to support a set of general, straightforward, and universally-applicable recommendations for interventions that foster health system strengthening, aid coordination, and improved access to health services (22). It highlighted the evidence gaps around local perspectives, contextual factors, issues of accountability and legitimacy, and specific challenges within the international aid and development sector. Accordingly, an out-of-the-box approach to link research with practice could help overcome the challenges of insecurity and instability of crisis-affected settings in which research occurs—for instance, best practice documentation could serve as a basis on which to build the evidence (22).

Save the Children's FP and PAC programs operating in humanitarian settings may have contributed directly or indirectly to local health systems strengthening. The objective of this paper was to identify programmatic components that were perceived by stakeholders to be critical in contributing to health system strengthening in the humanitarian context of the DRC (North Kivu Province) and fragile setting of Somalia (Karkaar region of Puntland State).

## Materials and Methods

We applied a qualitative approach using semi-structured interviews and focus group discussions with key informants to gain insights into their views and perceptions related to the program contributions to health system strengthening.

### Framework

The Health System Building Blocks framework of the World Health Organization guided the design of the research instruments (23). The building blocks include service delivery; medical products, vaccine, and technology; information, learning and accountability; health workforce; leadership and governance; and financing. As awareness-raising is essential for community mobilization, we expanded the six building blocks to embed community under information, learning, and accountability, keeping in mind that the community is also at the center of service delivery (24).

### Participants and Location

Save the Children identified key informants through convenience sampling as allowed by time, availability of participants, and security considerations. Informants included representatives of the community, the health workforce at the MoH-managed health facilities, the district and provincial MoH, Save the Children program staff, and the United Nations Population Fund at the program location. Focus-group discussions of 4-10 participants were organized separately for the community, healthcare workers, and Save the Children staff and key-informant interviews with those holding a managerial position. The research covered the health zones of Karisimbi (Goma) and Mweso (Kitchanga) in DRC, and the cities of Garowe and Qardho in Somalia.

### Data Collection and Analysis

We developed the interview guide based on the WHO Health System Building Blocks framework. For the community, questions addressed the themes of access, coverage, quality, effectiveness, responsiveness, efficiency, and social and financial protection, which are areas directly influenced by the quality of the health system. At the end of the interview, community members and managers were invited to give their priority recommendations to improve Save the Children program and further its contributions to health system strengthening. The field team reviewed and tested the interview guide in English and Somali in Somalia and French and Swahili in DRC. Recommendations from pilot testing were consolidated into the final interview guide (see Supplementary Materials).

An independent evaluator (male) led the data collection with the support of a local interview team in each country. The teams were composed of Save the Children staff working in monitoring and evaluation as well as project management (three in DRC and two in Somalia, all male—at the time of the data collection, there was no female staff available to join the interview teams). The interviews were audiotaped after obtaining the agreement from participants.

Interviews occurred from 28 January to 6 February 2020 in DRC and from 11 to 18 March 2020 in Somalia. Table 1 summarizes the interview types by participant profile and gender. In DRC, there were 11 focus group discussions and 7 key informant interviews with a total of 21 female and 53 male participants. In Somalia, there were seven focus group discussions and four key informant interviews with a total of 33 female and 19 male participants. In total, women accounted for 43% (54/126) of all respondents.

**Table 1:** Interview types by location, participant profile, and gender in the Democratic Republic of Congo and Somalia.

**Location**	**Participants**	**Gender**		**Total**
		**Female**	**Male**	
**DR Congo—focus group discussions**				
Karisimbi, Virunga General Hospital	Satisfied clients	3	0	3
Karisimbi, Save the Children office in Goma	Save the Children staff	2	5	7
Karisimbi, Virunga General Hospital	Men of light (male champions)	0	5	5
Karisimbi, Mugunga	Youth female peer educators (all unmarried) (18-20 years)	6	0	6
Karisimbi, Mugunga	Youth male peer educators (all unmarried) (17-18 years)	0	4	4
Mweso health center	Nursing staff	0	7	7
Mweso health zone	Men of light	1	5	6
Mweso health zone	Youth peer educators (18-23 years)	5	7	12
Mweso health zone	Religious leaders	0	4	4
Mweso health zone	Community intermediaries	0	2	2
Mweso health zone	Community intermediaries	2	5	7
**DR Congo—key informant interviews**				
Karisimbi, National Adolescent Health Program	Lead	0	1	1
Karisimbi, Virunga General Hospital	Medical director and head nurse for FP-PAC	1	1	2
Karisimbi, National Reproductive Health Program	Lead and co-lead	1	1	2
Karisimbi, Health Zone Central Office	Medical director and supervising nurse	0	2	2
Karisimbi, Pole Institute	Director	0	1	1
Karisimbi, United Nations Population Fund	Reproductive health and supply chain manager	0	1	1
Mweso, Health Zone Central Office	Medical director and head nurse	0	2	2
	Total, DR Congo	21	53	74
**Somalia—focus group discussions**				
Gaanlibah maternal and child health center	Providers	6	0	6
Shabelle maternal and child health center	Providers	6	0	6
Gaanlibah maternal and child health center	Mothers	6	0	6
Gaanlibah maternal and child health center	Young women (three unmarried) (17-21 years)	6	0	6
Gaanlibah maternal and child health center	Fathers and husbands of users	0	9	9
Shabelle maternal and child health center	Unmarried young men (20-28 years)	0	6	6
Save the Children field offices	Staff	2	3	5
**Somalia—key informant interviews**				
Gaanlibah maternal and child health center	Head	2	0	2
Gaanlibah maternal and child health center	Health district officers	2	1	3
Shabelle maternal and child health center	Head	1	0	1
Central Ministry of Health	Officials	2	0	2
	Total, Somalia	33	19	52
	Grand total	54	72	126

After transcription and translation into French (DRC data) and English (Somalia data), the data was single-coded and analyzed thematically using QSR NVivo 12 software. A basic codebook that described all the nodes was established and used to code data. The health system building blocks served as the framework of analysis. The codebook was enriched with new emerging nodes during the coding process. Themes were compared across the groups to explore similarities and differences, and we interpreted and presented the data using the participants' words as illustrations. To ensure the validity of the analysis and interpretation, key informants reviewed earlier drafts, and the final document incorporated their feedback.

### Ethics Approval and Consent to Participate

The Western Institutional Review Board determined that this evaluation did not constitute research and offered an IRB exemption (22 January 2020). However, we sought and obtained local approval in DRC through the Université Libre des Pays des Grands Lacs and in Somalia through the Ministry of Health. The Ethics Review Committee of Save the Children also approved the protocol. Additionally, all participants provided informed consent, and transcriptions did not record any names or information that would compromise participants' anonymity.

## Results

The analysis of the qualitative results suggests that the initiative contributed to strengthening different health system building blocks in both countries. Most importantly, community members overwhelmingly reported benefits of the program on the health of mothers and children as well as positive socio-economic impacts. For instance, such impacts were decreased home expenditure on children's nutrition and care or pregnancy prevention through informed voluntary contraception, which allowed women to study or work. Notably, the program appeared to have contributed to lifting a negative veil of misconceptions and fears surrounding contraception.

*In my opinion, the big change is the fact that, unlike in the past, our parents gave birth to up to fifteen children. This posed a great problem in caring for their children, especially during the period of recurring wars in our country. Today, thanks to this program brought by Save, everyone already knows how to do family planning and take care of their children properly. There are no more cases of malnutrition. –* Male community member, DRC

### Governance

#### Policies and Guidance

According to many participants from both countries, the initiative played a role in advocating for and positioning PAC on the national agenda along with FP. As a result, various policy documents integrated FP and PAC over the years (see Box 1).

Box 1 **Examples of programmatic influences on policy changes.**

- Task-sharing of manual vacuum aspiration to midlevel providers (nurses and midwives) and allowing manual vacuum aspiration in primary healthcare facilities;
- Task-sharing of long-acting and reversible contraceptive services to midlevel providers and allowing the availability of implants and intrauterine devices in primary healthcare facilities;
- Advocating for the abolishment of informal “couples counseling” obligations, where using contraception required the husband written or in-person permission;
- Expanding health management information system tools of the Ministry of Health to disaggregate data by new or returning user, contraceptive method, evacuation method, and age;
- Advocating for more favorable policies for adolescents to access sexual and reproductive health services.

In DRC, participants, from male champions (“men of light”) to decision-makers underscored the importance of the Law No. 18/035 of 13 December 2018, which they perceived as a game-changer as it allowed every individual of reproductive age, and therefore adolescents, “after informed consent to benefit from a contraceptive method.” Furthermore, the will of the woman or girl takes precedence over her husband/partner's opinion. As a staff member of Save the Children put it, the program “*has awakened the Congolese Government to the needs of women to let them decide.”*

*We had received many threats for having given methods to certain women because here, at home, it's the man who decides. But thanks to the partner [Save the Children], the new law now stipulates that it's the woman who has to decide about her health*. – Health facility manager, DRC.

Participants reported that this would not have been possible without Save the Children and partner organizations as they had strategically engaged with and advocated to the MoH and provincial authorities in addition to training relevant staff members on FP, PAC, and SRH more generally. In both countries, such capacity building likely impacted policies and practices (Box 1), and training materials developed in the context of the project were used as guidelines by the government.

#### Coordination

In both countries, Save the Children appeared to be actively engaged in coordination mechanisms with the government and other actors. For example, participants from the MoH reported how Save the Children staff, through the initiative, had been active and systematically engaged in coordination mechanisms at the provincial and health zone levels, including co-chairing working groups on FP. Staff participated in monthly coordination meetings and were reported to be quick in responding to needs related to FP and PAC services, such as addressing contraceptive stockouts or facilitating supportive supervision, even in facilities that were not part of the initiative.

Notwithstanding the similar contributions to strengthening coordination in both countries, the MoH participant in Somalia offered insights on ways to make such meetings not only technical but also political—an advocacy platform for political buy-in.

*It seems the coordination is only specific to the technical level personnel and the program needs political commitment in some parts. So, I would recommend including political figures from the parliament and ministerial level to have increased commitment. The religious leaders' meetings are held in Qardho and Garowe, where many people don't have access to. So I would recommend to make this meeting a regional or district level giving access to more people and getting new ideas*. MoH participant, Somalia

### Health Workforce

In both countries, Save the Children, in partnership with the MoH, established in Somalia and supported the establishment in DRC of a training center to become a hub for capacity building in clinical care. Stakeholders perceived it to be a highly strategic investment. Backed by FP and PAC champions, who played the role of master trainers, and adequate training materials, including anatomical models and competency-based curricula, these structures contributed to the capacity development of project staff as well as personnel from other health structures backed by the government or different health partners. Save the Children and the MoH also supported the champions to extend training and supportive supervision work beyond Save the Children to “*make sure that the project is sustainable because of the capacity building of staff at every level*,” as reported by a Save the Children participant. For example, in DRC, this inclusive strategy had benefited outreach facilities within the Virunga General Hospital coverage area and the northern areas of the province. There, geography and insecurity had hampered access and regular program provision.

Participants overwhelmingly reported how the capacity development workshops had adopted a state-of-the-art competency-based training approach using anatomical models and checklists and underscored the usefulness of post-training supportive supervision visits made jointly by Save the Children and government staff. As a result, providers reported improved competencies as well as increased confidence, as shared by participants in Somalia:

*The training gave us the confidence to do our job. The training lifted our reputation thanks to the good job we do for our patients because our work reflects the good training we received*. – Provider, Somalia

### Supplies

Participants with programming roles stressed the important contribution of Save the Children's model for supply chain management, one that is characterized by reactivity and reliability— “With Save, we see action. There are other partners who wanted to do the same activity, but we did not feel their approach as with Save the Children,” as reported by the Health Zone Central Bureau in DRC. Training workshops on supply chain management with reporting and other logistic management tools benefited both project staff as well as personnel from the MoH, underscoring again the potential legacy of the project to the health system.

*The central warehouse in Garowe run by the ministry just told us of the impact the supply management system had on their reporting, recording, and requesting for supplies as well as monitoring the stocks. In 2017, we sent one of our staff to Bari to train government staff and now we are planning to send him to Mudug to train their supply chain officers and provide them with tools*. – Save the Children staff, Somalia

Critically, the supply management approach resulted in no stockout down to outreach areas, as reported by community volunteers in Kitshanga. In fact, Save the Children was recognized to have contributed to the delivery of supplies and products all the way toward the “last mile,” i.e., to help these reach health facilities. However, contraceptive security was reported to remain fragile in both Somalia and DRC due to the inadequate in-country availability of supplies, the time lag to obtain supplies from national and international supplies, and the dependency on donors to procure supplies.

### Financing

Through the initiative, FP and PAC services and contraceptives were free of charge to users at the point of care, which removed a significant barrier to utilization. Participants mentioned that the MoH has been slow in committing a budget for the purchase of contraceptives. However, participants widely warned against the risks posed to the uptake of contraceptives, including by young people, once Save the Children withdraws its support to the program. For participants, the MoH is far from being ready to take over the project in terms of removing fees for services and contraceptives, which may negatively impact service utilization by adolescents and young people.

*If the partner withdraws when the government is not yet in a position to provide contraceptives to the population, this will be a barrier to young people! Imagine a young person arrives at the service to be given a prescription; she may not go and fill it if she cannot afford it. –* Adolescent Health National Program staff, DRC

In DRC, some participants reported how community health insurance schemes seemed to be working in stable areas where development programs were feasible, such as in the neighborhood of the Virunga General Hospital. Such insurance schemes could reduce the financial barriers that affect the population's access to FP and PAC services. However, it was reported that for now, they covered mainly sickness conditions and childbirth but not contraception as this was considered as health promotion.

### Information

#### Demand Generation

In both countries, participants reported the multiple channels used by the project to reach the community and raise awareness about the importance of FP and PAC. The information and positive messaging likely had an impact beyond the project coverage zones.

For example, in DRC, communication channels included radio messaging (see Box 2), men of light, satisfied women, and community volunteers. Peer educators and community volunteers received support from the initiative but managed independently and would continue to do so after the end of the project.

Box 2 **Messaging on the radio in the Democratic Republic of Congo.**

The initiative's messages on family planning and postabortion care may have reached more than 10 million listeners, according to the director of the Pole Institute Radio (*pole*: compassion in Swahili), a non-profit organization dedicated to peacebuilding through inter-cultural understanding. According to the Institute, Pole covers 90% of the territory in North Kivu, 60% in South Kivu, 40% in Lituri, 25% in Uganda, 15% in Rwanda. Listeners were reported to appreciate the “Save the Children Song” as they regularly asked the radio to play it.

Building on the initial approach, which broadcasted the song in a linear one-way direction toward the listeners, more interactive options could be explored in the future, including:

- Communicating a unique phone number or weblink at the end of the radio message, so that listeners could provide feedback. The Pole Institute has algorithms to analyze the quantitative and qualitative impact of auditors' feedback;
- Adopting discussion spaces, such as *Forum for popular expression* or *Listeners' club*, where different members of the community can interact with a clinical expert;
- *Audio series* at the end of which auditors could call and leave comments and questions that would be directly addressed by an expert.

In Somalia, Save the Children staff reported the difficulty in implementing family planning programs due to conservative traditions and practices— “even some other NGOs failed.” The successful buy-in came from the involvement during the pilot phase of women from the community who championed the cause and paved the way for the next stage of the project. Equally important was the engagement of religious scholars, elders, and community leaders who advocated for FP during community mobilization campaigns and meetings with the MoH. Father-to-father, mother-to-mother, and young people-to-young people sessions were reportedly also critical in raising awareness. However, for many, more could be done, especially reaching young people, men, and those living in remote rural settings.

#### Health Information System

In both countries, participants with program management functions reported the importance given by Save the Children to the use of data to inform decision-making and action, such as facility-based charts as a reflection tool during supportive supervision visits and discussions with providers or the review of routine data with the MoH and community stakeholders. Participants also highlighted the novelty of age-disaggregated data for adolescents and young people, which Save the Children introduced in 2017.

For example, in Somalia and DRC, providers who are the first line data collectors and users reported as follows:

*Before, reports were stored in a facility, and, if needed, they have to be dug from a pile of papers. Now all the data is contained in the wall charts depicting the whole year. This has simplified the reporting process…It is very different from other programs. Family planning has age category to identify mothers younger than 18 years of age since those are the higher risk group. It is also different in that every provider sees how many mothers he/she did serve*. - Provider, Somalia

However, they mentioned the fragmentation and potential data collection burden induced by the different reporting needs of various NGO-supported projects and donor requirements.

### Health Services

Participants in both countries were of the view that thanks to the project, FP and PAC services were of quality in addition to being free of charge at the point of care. In DRC, participants highlighted that the FP and PAC services reinforced women's choice to decide on their own. The gratuity of services was instrumental in service uptake, including the adoption of long-acting contraceptives among new users. Interestingly, the perspectives of men reflected the transformation brought by the program in terms of access, availability, and removal of service fees supported by the program. The high quality of services and particularly the availability of postabortion care in the project facilities benefited the health system.

*Before, there were people from rural areas who used to die due to loss of blood but now they are brought to the centers, which are open at all times. Birthspacing was something that we needed because if the children are not spaced, the mother suffers from malnutrition*. - Male community participant, Somalia

Additionally, providers and master trainers outside the project trained on FP and PAC through the project contributed to expanding the coverage of these services within the health system beyond the facilities supported by Save the Children. As a result, there was an increased number of reference centers for FP and PAC thanks to the initiative, as reported by the MoH from Somalia.

Female peer educators in DRC were enthusiastic about the emphasis on adolescents and young people and their roles in facilitating access to information and services to this segment of the community. However, they mentioned angrily instances where providers or pharmacists were not respecting the confidentiality and privacy of adolescent clients by informing their family or parents.

*I also think it's punishable by law: a good doctor or nurse is not allowed to disclose clients' medical information…So, I think that when you educate us, you must also do the same for nurses because apparently there are a few who have no medical ethics. –* Young female community participant, DRC

**Figure 1:**
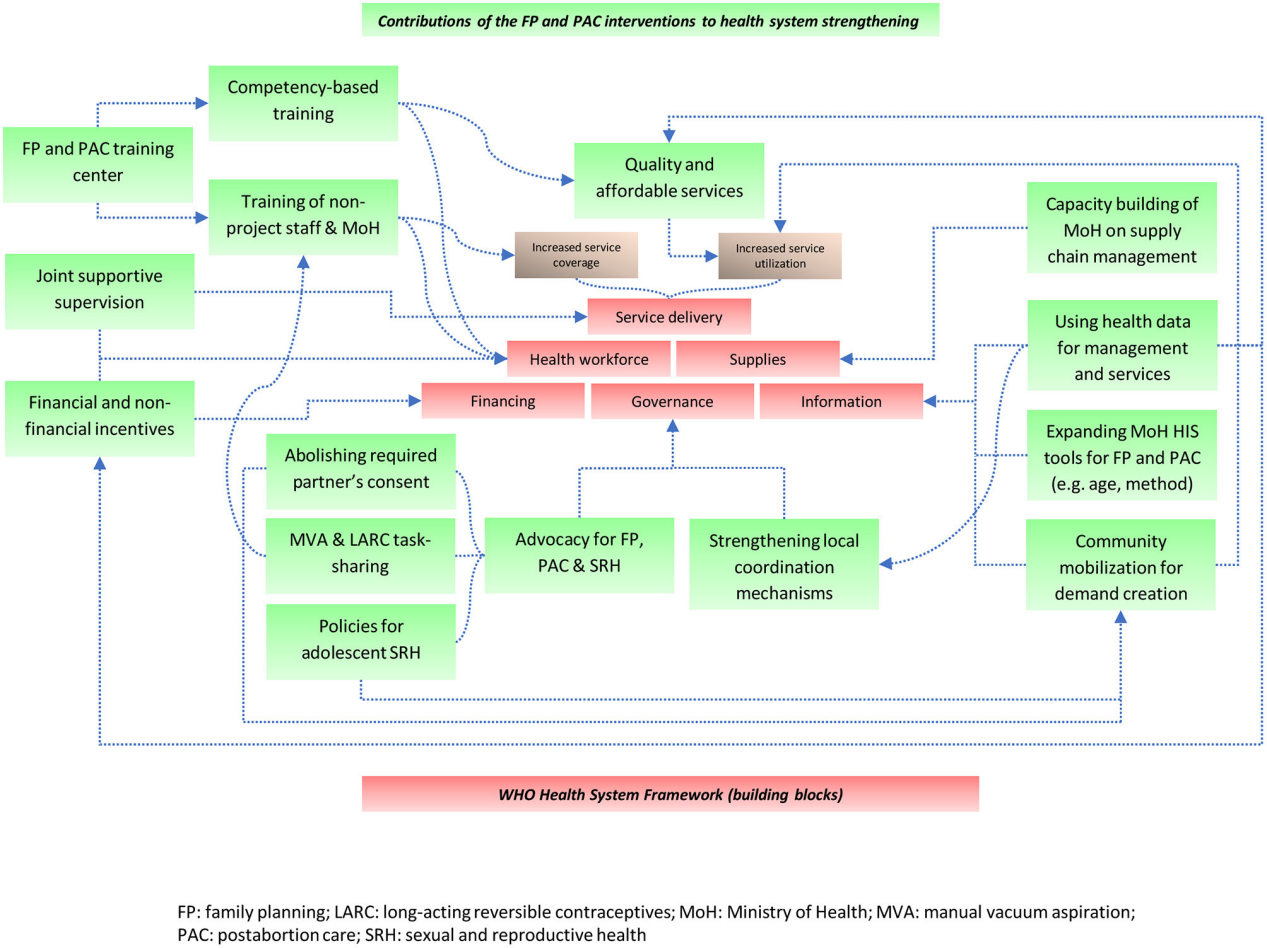
Strengthening health systems in humanitarian settings: a contribution model from family planning and post-abortion interventions in the Democratic Republic of Congo and Somalia.

Save the Children staff in both countries acknowledged the need to strengthen adolescent and young people-centered services not only within but also beyond the FP and PAC project. Other recommendations from participants are described in Box 3. Building on the participants' perspectives, Figure 1 summarizes the way FP and PAC program interventions were perceived to have contributed to strengthening health systems.

Box 3 **Recommendations from participants to improve FP and PAC health services and contributions to health system strengthening.**

Participants from both countries seemed to agree on the need to:

- Ensure the continuity and expansion of free FP and PAC services by advocating against the donor's withdrawal;
- Strengthen the integration of FP and PAC into primary healthcare services;
- Continue to ensure the continuous availability of supplies;
- Further train, support, and retain healthcare staff on FP and PAC;
- Continue focusing on adolescent-inclusive services and increasing competencies of all staff dealing with the topic;
- Keep engaging and mobilizing the community, with a focus on men, adolescents, and hard-to-reach communities;
- Advocate to health ministries and like-minded partners to join efforts on FP and PAC programming and support enabling policy changes.

## Discussion

The multi-perspective results from community members to policymakers in both countries indicate that health programs in humanitarian settings, such as Save the Children's FP and PAC initiative, could contribute to strengthening health systems. This was accomplished by positively influencing national policies and guidance, strengthening local coordination mechanisms, capacitating the healthcare workforce with competency-based training, mentoring, on-the-job training, and supportive supervision (benefiting facilities supported by the project and beyond), developing the capacity of project and MoH staff in the effective management of the supply chain, actively and creatively mobilizing the community to raise awareness and create demand, and providing quality and affordable services. Contributions of this package to increased utilization of services and long-acting contraceptives were quantitatively evidenced in previous publications (12–14).

Save the Children's strategic and programmatic investments align with the results of a study published in 2017 by Martineau et al. who analyzed research evidence on rebuilding health systems in conflict- and crisis-affected countries (25). According to the analysis, the starting points for policy development and systems strengthening are the community, human resources for health, and institutions.

With regard to institutions, the arrival of multiple actors and resulting power dynamics requires strong coordination to optimize the capacity building and response of national and local institutions (26). The FP and PAC initiative devoted resources toward coordination that drew from information and results gathered at the facility level. Local MoH representatives, program staff, and facility managers regularly reviewed together incoming data and collaborated in real-time to find solutions where needed. Strategically engaging local leaders and developing the capacity of local MoH representatives likely helped enhance their ownership of the program and accountability toward the community, while buttressing the legitimacy of FP and PAC services.

At the community level, understanding the impact of crises on households' resilience and access to care, including the ability to pay for services at the point of care, which could be compromised for years in countries under stress, is essential for developing responsive interventions and policies (27). Save the Children carefully examined community barriers that impeded demand for and access to services before crafting its FP and PAC interventions. The program prioritized societal partnerships and collaborated with various community stakeholders and champions to help mobilize the population through small group sessions (24). These sessions tackled stigma around postabortion care and raised awareness about FP and PAC services availability. Community champions included religious or community leaders, women who were satisfied with the services they received, champion male partners, satisfied couples, young female and male peer-to-peer counselors, and community volunteers and health workers. In addition to these efforts, the initiative might have further advanced the critical role of the community in strengthening systems for health by reinforcing social behavior change approaches and integrating community-based services as appropriate.

As for human resources for health, the fragmentation of remuneration and incentive packages is common in humanitarian settings (28), and adequate support for healthcare staff is critical to ensure a balanced distribution across gender, sectors, and geography (29, 30). The FP and PAC initiative invested heavily in this aspect—with apparent success in the short term but the MoH needs to take the lead to adequately pay its health workers in the long term. The investment in human resources for health through training centers and continuous coaching and supportive supervision likely had a multiplier and potentially long-lasting effect as capacity development efforts were extended to central and zonal government staff as well as service providers from areas that were not directly supported by Save the Children. The program actively pursued a strategy that consisted of increasing the knowledge and skills of local staff, involving them in identifying and implementing solutions to new problems and motivating them by ensuring that health facilities are adequately equipped, topping up inadequately low salaries, and demonstrating visible improvements in quality of care and service volume. All these components are known to improve the performance of human resources for health in low-income countries (31).

By way of example, policy change is the result of a complex process, where human agency and, more specifically, the mobilization of specific actors involved in the policy process constitute a key factor to drive change (32). The multiyear funding for the initiative allowed Save the Children to have, over the past decade, a core group of Congolese and Somali staff dedicated to leading, managing, and delivering on the FP and PAC program. The government and community informants appeared to have appreciated their ongoing engagement in program and policy dialogue with health officials, their reliable presence in coordination meetings, supportive supervisions, and willingness to support the MoH at different levels and respond to their calls for action in a consistent and solution-oriented approach. The dynamic presence and added value of Save the Children's project staff, Congolese and Somali nationals, may have influenced the view that the project contributed to strengthening health governance.

The volume and quality of services generated by the program were the outcomes of careful program design and implementation, which accounted for several quality improvement processes. On the supply side, these processes focused on removing FP and PAC service fees, establishing a procurement system that secured the continuous availability of a mix of short-acting and long-acting contraceptives and supplies (manual vacuum aspiration kits, misoprostol for PAC, analgesia, antibiotics, materials for infection prevention and control, intrauterine device and implant insertion and removal, and job aids, among others), capacitating service providers through competency-based training, training staff—including the MoH personnel—on supply chain management and using health data to inform decision-making at the management and facility levels. There is evidence that such quality improvement practices can play a potential role in health system strengthening in low-income and middle-income countries (33, 34).

Despite the achievements of the initiative in meeting the FP and PAC needs of clients and contributing to health system strengthening, participants' overall impression was that the health system was not yet ready to take over the entirety of the program. Participants overwhelmingly shared the view that health facilities could not yet offer services and contraceptives that are free of charge to users.

After close to 10 years of financial and human resource investment in the project, it is essential to reflect on the reasons why the system is not yet prepared to take over FP and PAC services fully. In DRC, the health expenditure has remained mostly stagnant at approximately 4% of the gross domestic product between 2010 and 2017, according to the World Health Organization Global Health Expenditure Database (35). With few resources and investments from the MoH, most of the health programs and facilities in DRC have been relying on external sources of support (2). In North Kivu, cycles of violence and insecurity combined with Ebola outbreaks have affected the development and ripening of a functional and sustainable health system and primary health care services (36). In Somalia, there is no recent WHO data on health expenditure—in 2002, the figure was 2.6%. In 2018, the Somali government reported a budget of USD 1.4 million (0.5% of the national budget) for “protecting public health” (37).

Overall, the need for quality FP, PAC, and SRH services of communities living in humanitarian and fragile settings continues to be high in DRC, Somalia, and countries with similar contexts. The COVID-19 emergency preparedness and response measures in these settings may negatively impact already limited public health resources and overburdened health systems. This could further compromise the population's timely access to these services with potentially dire consequences on the prevention of unplanned pregnancies and the management of abortion complications (38). Experience from the Ebola outbreaks in West Africa and currently in eastern DRC has shown a sharp reduction in access to SRH services during these epidemics (39, 40). Therefore, lessons learned from the initiative over the past decade and previous epidemics combined with the threats due to COVID-19 to maintain access to services, including the MISP, dictate that donors and local and global stakeholders must urgently commit additional resources to sustain health systems and *essential services, such as FP and PAC*.

### Limitations

The qualitative evaluation faced several limitations, including possible translation mistakes, non-representative sampling, and social desirability bias. Regarding sampling, there were more male than female participants in DRC despite efforts from the research team to reach a balanced gender representation. Reasons may include the fact that we held more key informant interviews with participants with leadership functions in DRC than in Somalia and that men hold most of the leadership positions in the community and among local health authorities. However, we ensured that women could speak for their own voices by stressing the confidential and anonymous nature of the discussions and strived to represent their views and perspectives in the report. Additionally, participants may have provided the responses they believed the facilitators desired, although all efforts were made to have an independent evaluator in both settings despite insecurity and timing constraints. Social desirability bias could be further compounded by the fact that the local interview teams were all male in both contexts. Finally, although Save the Children staff had no role in the data analysis, data collection was mostly done by its staff, which could be a source of bias. However, an independent evaluator was recruited to design and oversee the evaluation to ensure objectivity. Recruiting an entire team of independent researchers would have maximized objectivity but was not feasible due to limited budget.

### Conclusions

Addressing the SRH needs of crisis-affected populations with programs that contribute to long-term development exemplifies the tensions within the humanitarian-development nexus. Feedback from a range of stakeholders indicates that an intervention model, such as the one designed by Save the Children and local MoH partners, was effective in enhancing access to high-quality health services in humanitarian and fragile settings while contributing to strengthening several components of the health system. As showcased in this qualitative research, essential interventions, such as FP and PAC, must be considered as indispensable components of health services that do not strain but could contribute to strengthening health systems in humanitarian settings.

## Data Availability Statement

The raw data supporting the conclusions of this article will be made available by the authors, without undue reservation.

## Ethics Statement

The studies involving human participants were reviewed and approved by Western Institutional Review Board Université Libre des Pays des Grands Lacs Ministry of Health in Somalia. The patients/participants provided their written informed consent to participate in this study.

## Author Contributions

NT, JM, and RA conceived the study with the contributions from BM, AS, EM, CM, MG, and VJ. NT and HH collected the qualitative data with the support of BM, JC, J-BM, PL, AS, MAl, MAr, JD, MK, JA, and BG. NT, HH, and NP analyzed the qualitative data with the contributions from RA, JM, BM, AS, EM, CM, MG, and VJ for the interpretation. NT drafted the initial manuscript. All authors contributed to manuscript revision and have approved the final version.

## Conflict of Interest

The authors declare that the research was conducted in the absence of any commercial or financial relationships that could be construed as a potential conflict of interest.

## Publisher's Note

All claims expressed in this article are solely those of the authors and do not necessarily represent those of their affiliated organizations, or those of the publisher, the editors and the reviewers. Any product that may be evaluated in this article, or claim that may be made by its manufacturer, is not guaranteed or endorsed by the publisher.
