# Supplementary material for: Strengthening Health Systems in Humanitarian Settings: Multi-Stakeholder Insights on Contraception and Postabortion Care Programs in the Democratic Republic of Congo and Somalia
Source: Front Glob Womens Health. 2021 Aug 30;2:671058. doi: 10.3389/fgwh.2021.671058 (PMC8593961; doi:10.3389/fgwh.2021.671058)
Supplement: Supplementary file 1 [file Data_Sheet_1.pdf]

Qualitative interview guide

Participant:

Instructions: *participants will be identified by Save the Children.*

ID code	# participants	Location	Date (dd/mmm/yy)<br>__ __ / __ __ __ / 20	Start time<br>__ __ : __ __	End time<br>__ __ : __ __	Facilitator initials

TO READ AT THE BEGINNING

Introduction
Welcome to this interview. We are delighted that you were able to save some of your time to participate in this interview. We will begin by introducing ourselves. My name is ....(Toan, independent consultant)....and I will moderate the interview. In the team we have .... who will take notes.

Objective
Our evaluation (research) aims to understand better how the family planning / post-abortion care program of Save the Children has contributed to strengthening the health system in …(location)……..................... and identify recommendations on how the program can further strengthen the health system. This will help improve Save the Children's current and future programs and eventually better meet the family planning and post-abortion care needs of women, girls and couples.
The discussions will be recorded and then destroyed once the transcript is completed. All information provided will be stored securely and confidentially and will not be passed on to third parties. No real names will be used in reports resulting from the information collected. All information will be treated confidentially.

Explain the basic rules for the interview
This is a friendly interview, so there is no right or wrong answer. We encourage you to relax and not hesitate to express your opinions. Participation is free and voluntary. We will propose a series of open-ended questions or topics for discussion and you can begin to answer. As a reminder, you do not have to answer all of our questions, and you can skip questions. Please keep your mobile phone silent so as not to disrupt the discussion. We will spend approximately one hour in our interview (and refreshments will be served to you at the end of the interview).
We have reviewed your consent form, which describes the evaluation study in detail and gives us permission to talk to you. As a reminder, we will be using a digital recorder to record our conversation.
Do you have any questions before we begin the interview?

Moderator: Turn on the digital recorder: *"I am (NAME OF MODERATOR) and am conducting the interview (DETAILED INTERVIEW IDENTIFICATION CODE) on [DATE] [TIME OF BEGINNING]".*

	Main questions	Probing questions
**Strengthening the health system**		
1.	*[Service delivery]*<br>What are the contributions of SC FP/PAC programs in terms of reinforcing the overall **delivery of quality health services**?	a. Quality of care dimensions<br>- Safety of methods and services<br>- Respectful<br>- Acceptable, person-centered, as per the person's preferences/norms<br>- Confidential<br>- Free choice<br>- No-discrimination<br>- Availability of services when individuals need it<br>- Continuity of care<br>b. Recommendations for further improvements and sustainability beyond the program period?
2.	*[Supplies]*<br>What are the contributions of SC FP/PAC programs in terms of reinforcing the overall **supply chain of medical products and commodities**?	a. Availability of commonly preferred method mix?<br>b. Quality and safety of contraceptives?<br>c. Availability of key supplies (specula, pregnancy tests)?<br>d. Availability of product and stockout in outreach/harder to reach areas?<br>e. Recommendations for further improvements and sustainability beyond the program period?
3.	*[Community/Demand creation/Information]*<br>What are the contributions of SC FP/PAC programs in terms of reinforcing the overall **creation of demand for services**?	a. How was the community involved in designing the program?<br>b. Reaching adolescent, unmarried, disabled, and other priority populations?<br>c. Engaging men?<br>d. Engaging decisionmakers in the families (e.g., stepmothers)?<br>e. Support from religious authorities?<br>f. Support from local leaders?<br>g. In-school/at work programming?<br>h. Voucher programs?<br>i. Recommendations for further improvements and sustainability beyond the program period?
4.	*[Health workforce]*<br>What are the contributions of SC FP/PAC programs in terms of reinforcing the overall **health workforce**?	a. MOH staff?<br>b. SC staff?<br>c. Skilled staff at outreach/harder to reach facilities?<br>d. Workload compared to remuneration/social benefits/career opportunities?<br>e. Supportive supervision and accountability mechanisms?<br>f. Remaining gaps in training needs?<br>a. In-service<br>b. Pre-service<br>g. Recommendations for further improvements and sustainability beyond the program period?
5.	*[Governance/Policy & guidance]*<br>What are the contributions of SC FP/PAC programs to influencing subnational / national **policies and guidances**?	a. Nature of policy and guidance?<br>b. Dissemination and implementation of policy and guidance?<br>c. Recommendations for further improvements and sustainability beyond the program period?

6.	*[Governance/Coordination]*<br>What are the contributions of SC FP/PAC programs in terms of strengthening **coordination with key stakeholders**?	a. MOH *(note: MOH main partner in Somalia)*<br>b. NGOs *(note: ask in particular in Somalia – DRC had several local partners)*<br>c. United Nations agencies<br>d. Community leaders<br>e. Recommendations for further improvements and sustainability beyond the program period?
7.	*[Health information system]*<br>What are the contributions of SC FP/PAC programs in terms of **strengthening the health information system?**	a. Locally<br>b. In the province<br>c. Nationally<br>d. Is there a reliable HIS capturing FP/PAC indicators and service performance?<br>e. Is data used to improve the quality of FP/PAC services/programs?<br>f. Recommendations for further improvements and sustainability beyond the program period?
8.	*[Financing]*<br>What are the contributions of SC FP/PAC programs in terms of **strengthening investment in FP/PAC services?**	a. From local/provincial/national authorities<br>b. From international partners<br>c. Expenses from commodities supply?<br>d. Expenses from service delivery?<br>e. Client-side co-payment/expenses?<br>f. Other financing mechanisms? Voucher programs? *(note: PBF in the DRC by the WB, which is a source of operational confusion)*<br>g. Recommendations for further improvements and sustainability beyond the program period?
**Conclusion**		
9.	In summary, what would be your priority recommendations (max 3) to improve or help with the sustainability of this program?	
10.	Before we conclude, do you have anything else to share with us on the SC FP/PAC programs and services?	

It's the end of our discussion. Thank you for your time.

Questions for the focus group discussions with the community

Perception of achievements and outcomes related to SC FP/PAC programs and services		
11.	*[Access]*<br>What do you think of the SC program in terms of improving **access to FP/PAC services**?	a. What was the situation before the program?<br>b. What has the program achieved in terms of (bold theme)?<br>c. Why?<br>d. Affordability of services?<br>e. Harder to reach, poor, underserved, marginalized, socially-excluded, young, unmarried, disabled, LGBTI individuals/communities?<br>f. Men and male adolescents?
12.	*[Coverage]*<br>What do you think of the SC program in terms of improving **coverage for FP/PAC services**?	a. What was the situation before the program?<br>b. What has the program achieved in terms of (bold theme)?<br>c. Why?<br>d. Harder to reach, poor, underserved, marginalized, socially-excluded, young, unmarried, disabled, LGBTI individuals/communities?
13.	*[Quality]*<br>What do you think of the SC program in terms of improving the **quality of FP/PAC services**?	a. What was the situation before the program?<br>b. What has the program achieved in terms of (bold theme)?<br>c. Why?<br>d. Quality of care dimensions<br>- Safety of methods and services<br>- Respectful<br>- Acceptable, person-centered, as per the person preferences/norms<br>- Confidential<br>- Free choice<br>- No-discrimination<br>- Availability of services when individuals need it<br>- Continuity of care
14.	*[Effectiveness]*<br>How would you describe the **improvement of the health status** of women/the community as a consequence of SC FP/PAC services?	a. What was the situation before the program?<br>b. Nature of health improvement?
15.	*[Responsiveness]*<br>How **responsive** were the services to women's/the community needs in terms of FP/PAC?	a. What was the situation before the program?<br>b. Examples of responsiveness to women's/community needs
16.	*[Efficiency/equity]*<br>What do you think about the **resources invested** in SC FP/PAC services?	a. Would you have invested resources differently? In other priorities? If so, what are they?<br>b. Sustainability of the program?<br>c. Integration with other services?
17.	*[Social/financial protection]*<br>What do you think about the **social or financial advantages** brought to women/the community as a consequence of SC FP/PAC services?	a. What was the situation before the program?<br>b. Examples?
18.	For certain categories of participants:<br>*[Rationale]*	a. What are the main difficulties you have encountered in this role?<br>b. What would help you improve your work?

	What motivated you to become... *[peer educators, etc.]*?	
**Conclusion**		
19.	In summary, what would be your priority recommendations (max 3) to improve or help with the sustainability of this program?	
20.	Before we conclude, do you have anything else to share with us on the SC FP/PAC programs and services?	

Notes
